# Latent Cardiometabolic Phenotypes and Their Sociodemographic Correlates Among US Adults: A Cross‐Sectional Latent Class Analysis Using NHANES (2011–2018) Data

**DOI:** 10.1002/dmrr.70225

**Published:** 2026-08-31

**Authors:** Greeshma Unnikrishnan, Abhinav Singh, K. R. Harijith

**Affiliations:** ^1^ Department of Dentistry, Regional Training Centre for Oral Health Promotion (M.P) and Oral Health Data Bank (M.P) All India Institute of Medical Sciences (AIIMS) Bhopal Madhya Pradesh India; ^2^ Department of Community and Family Medicine All India Institute of Medical Sciences (AIIMS) Bhopal Madhya Pradesh India

**Keywords:** cardiometabolic phenotypes, cardiometabolic risk, latent class analysis, metabolic syndrome, NHANES, socioeconomic determinants

## Abstract

**Aim:**

The study aims to identify distinct cardiometabolic phenotypes based on multiple metabolic risk indicators, assess their associations with sociodemographic characteristics, and evaluate income‐related differences through cardiometabolic risk using decomposition methods to quantify the contribution of observed factors to these disparities.

**Materials and Methods:**

A cross‐sectional analytical study was conducted using National Health and Nutrition Examination Survey (NHANES) 2011–2018 data. Adults aged 20 years and above with complete cardiometabolic biomarker data were included. Six cardiometabolic indicators including waist circumference, systolic blood pressure, fasting plasma glucose, glycated haemoglobin, triglycerides, and high‐density lipoprotein cholesterol were used to derive latent phenotypes using latent class analysis. Multinomial logistic regression examined associations between sociodemographic factors and phenotype membership. Oaxaca‐Blinder decomposition analysis assessed income‐related disparities in the high cardiometabolic risk phenotype.

**Results:**

A total of 7733 participants were included in the analysis. A three class model demonstrated optimal fit and identified metabolically healthy (54.9%), hypertension‐hyperglycemia (33.1%) and high cardiometabolic risk (11.9%) phenotypes. The high‐risk phenotype exhibited elevated probabilities of obesity, dyslipidemia and glycaemic abnormalities. Increasing age, male sex, lower income and lower educational attainment were significantly associated with high risk phenotype membership. Decomposition analysis demonstrated modest income‐related disparities, with waist circumference and education contributing the largest explained components.

**Conclusion:**

Cardiometabolic risk among U.S. adults clusters into three distinct phenotypes associated with sociodemographic and metabolic determinants. These findings highlight the need for integrated strategies targeting early metabolic dysregulation and broader social determinants of health.

## 1 Introduction

Cardiometabolic diseases, including type 2 diabetes and cardiovascular diseases, represent a major and growing global public health challenge contributing to morbidity, mortality, and significant healthcare burden. Global cardiovascular disease (CVD) burden has increased substantially over the past three decades. Between 1990 and 2019, the number of prevalent CVD cases nearly doubled from 27 to 523 million, while CVD ‐related deaths increased from 12.1 to 18.6 million [1, 2]. These conditions arise from a complex interplay of metabolic abnormalities such as central adiposity, dyslipidemia, impaired glucose metabolism, and elevated blood pressure, which frequently co‐occur within individuals. Increasing evidence suggests that these risk factors do not operate in isolation but tend to cluster, reflecting underlying pathophysiological and behavioural processes that contribute to disease progression [3, 4].

Emerging studies have demonstrated that cardiometabolic phenotypes are closely linked to broader social and environmental contexts. Cardiometabolic outcomes are influenced not only by biological factors but also by socioeconomic conditions, behavioural patterns, and structural determinants of health [5, 6, 7]. Importantly, disparities in cardiometabolic diseases are rarely attributable to a single risk factor but rather reflect the cumulative effects of multiple exposures operating across the life course. Socioeconomic status, particularly income, has been increasingly recognised as a critical determinant of cardiometabolic health, influencing access to healthcare, nutrition, and health promoting environments [7, 8].

Conventional approaches to cardiometabolic risk assessment have largely relied on single risk factors or predefined constructs such as metabolic syndrome. However, these frameworks impose rigid thresholds and may inadequately capture the heterogeneity of cardiometabolic risk within populations [9]. Increasing evidence suggests that individuals with similar levels of individual risk factors may differ substantially in their overall metabolic profiles and disease trajectories, highlighting the need for more nuanced, data driven approaches to characterise cardiometabolic heterogeneity [9, 10]. Latent variable methods have emerged as powerful tools to address this complexity. Early applications of factor analysis demonstrated that cardiometabolic variables may be organised into underlying domains such as glucose dysregulation and lipid metabolism, although findings have been inconsistent across populations [10]. More recently, latent class analysis (LCA) has been increasingly applied to identify distinct cardiometabolic phenotypes based on the co‐occurrence of multiple risk factors. These studies consistently demonstrate the existence of heterogenous subgroups, ranging from metabolically healthy individuals to high‐risk profiles, characterised by concurrent obesity, dyslipidemia, and hyperglycemia [11, 12, 13]. Importantly, such phenotypic classifications have been shown to provide greater clinical and epidemiological insight than traditional definitions by capturing real‐world clustering patterns.

Beyond biological clustering, cardiometabolic risk is strongly shaped by social and structural determinants of health. Socioeconomic position influences exposure to risk factors, access to healthcare, dietary patterns, and opportunities for physical activity, thereby contributing to disparities in cardiometabolic outcomes [7, 13]. However, most existing studies examining cardiometabolic phenotypes have focused primarily on identifying clusters, with comparatively limited attention to how these phenotypes are distributed across socioeconomic strata or the mechanisms of underlying observed disparities.

Addressing this gap requires an integrated analytical framework that combines phenotypic identification with inequality analysis. While regression‐based approaches can quantify associations between socioeconomic factors and phenotype membership, they do not explain the extent to which observed disparities arise from differences in measurable characteristics versus broader structural influences. Decomposition methods provide a complementary perspective by partitioning group differences into explained and unexplained components, thereby offering deeper insights into the drivers of health inequality [14, 15, 16].

The present study addresses these gaps by examining cardiometabolic phenotypes in a nationally representative sample of US adults from National Health and Nutrition Examination Survey (NHANES) cycles spanning 2011–2018. Specifically, this study aims to identify distinct cardiometabolic phenotypes based on multiple metabolic risk indicators, assess their associations with sociodemographic characteristics, and evaluate income‐related differences through cardiometabolic risk using decomposition methods to quantify the contribution of observed factors to these disparities.

## 2 Materials and Methods

### 2.1 Study Design and Data Source

This study is a cross‐sectional analytical study which utilised data from the National Health and Nutrition Examination Survey (NHANES) cycles spanning 2011–2018. NHANES is a nationally representative survey designed to assess the health and nutrition status of civilian non‐institutionalised population of the United States of America. The survey was conducted by the National Centre for Health Statistics (NCHS) using a complex multistage probability sampling design that combines interviews, physical examinations and laboratory assessments. Data from four NHANES cycles (2011–2012, 2013–2014, 2015–2016, and 2017–2018) were combined for the present analysis. Publicly available demographic, examination, laboratory, and questionnaire datasets were merged using the unique participant identifier (SEQN). Ethical approval for NHANES was obtained from the NCHS Research Ethics Review Board, and written consent was obtained from all participants. As this study utilised de‐identified publicly available data, additional institutional ethical approval was not required. This study was reported in accordance with the STROBE guidelines for cross‐sectional studies.

### 2.2 Study Population

Participants aged 20 years and older with complete data on cardiometabolic biomarkers were included in the analysis. After excluding participants with missing data on the variables of interest, the final analytic sample consisted of 7733 individuals.

### 2.3 Cardiometabolic Indicators

Six cardiometabolic indicators were selected based on their relevance to metabolic syndrome and cardiometabolic risk: (1) Waist circumference (BMXWAIST) (2) Systolic Blood Pressure (BPXSY1) (3) Fasting plasma glucose (LBXGLU) (4) Glycated haemoglobin (HbA1c; LBXGH) (5) Triglycerides (LBXTR) (6) High density lipoprotein cholesterol (HDL; LBDHDD). These indicators represent key physiological domains including central adiposity, glycaemic regulation, lipid metabolism, and blood pressure regulation.

### 2.4 Construction of Cardiometabolic Risk Indicators

For latent class analysis, variables were categorised into binary indicators based on established clinical thresholds. Central obesity was defined as waist circumference ≥ 102 cm in men and ≥ 88 cm in women. Elevated fasting glucose was defined as ≥ 100 mg/dL, elevated HbA1c as ≥ 5.7%, hypertriglyceridemia as triglycerides ≥ 150 mg/dL, low HDL cholesterol as < 40 mg/dL in men and < 50 mg/dL in women, and elevated systolic blood pressure as ≥ 130 mmHg. Systolic blood pressure was selected as the blood pressure indicator as it is a stronger predictor of cardiovascular morbidity and mortality than diastolic blood pressure (DBP), particularly among middle‐aged and older adults and is emphasised in contemporary hypertension guidelines. It also minimised the redundancy arising from the high correlation between SBP and DBP measurements [17]. Binary coding was applied (1 = normal; 2 = abnormal) to facilitate latent class analysis.

### 2.5 Latent Class Analysis (LCA)

Latent class analysis was conducted to identify distinct cardiometabolic phenotypes based on the six binary indicators. Models with three different number of classes were evaluated, and the three‐class model was selected based on model fit indices including Akaike Information Criterion (AIC), Bayesian Information Criterion (BIC), entropy, class interpretability and class size. Although the four class model demonstrated slightly lower AIC and BIC values, the three‐class model was selected as the optimal model due to higher entropy, better interpretability, and clinically meaningful phenotype structure. Participants were assigned to classes using maximum posterior probability. The identified classes were interpreted and labelled based on conditional probabilities of metabolic abnormalities within each phenotype.

### 2.6 Multinomial Logistic Regression

To examine the sociodemographic determinants of cardiometabolic phenotypes, multinomial logistic regression was performed using phenotype class as the outcome variables. Predictor variables included were: Age, Sex, Race/ethnicity, income to poverty ratio, and education level. Odds ratios (ORs) and 95% confidence intervals (CI) were calculated.

### 2.7 Income Inequality Decomposition

To complement the latent class analysis and multinomial logistic regression, Oaxaca‐Blinder decomposition analysis was performed to quantify the extent to which measured demographic, socioeconomic, anthropometric and behavioural characteristics explained income‐related differences in the prevalence of the high cardiometabolic risk phenotype. Decomposition analysis partitions observed differences between group endowments (explained differences attributable to variation in observed characteristics between income groups), coefficients (unexplained component which are the differences arising from variation in the effects of these characteristics between groups) and interaction component which is the combined contribution of differences in both characteristics and coefficients). The behavioural variables included in the decomposition model were smoking status and physical activity. Smoking status was obtained from the NHANES smoking questionnaire (SMQ020), which assesses whether participants had smoked at least 100 cigarettes during their lifetime. Participants answering ‘Yes’ were classified as ever smokers, whereas those answering ‘No’ were classified as never smokers. Physical activity was classified according to NHANES physical activity questionnaire (PAQ650), which asked whether participants engaged in vigorous‐intensity sports, fitness, or recreational activities for at least 10 min during a typical week. Participants reporting no vigorous recreational activity were classified as physically inactive, whereas those reporting participation were classified as physically active.

### 2.8 Statistical Analysis

All analyses were conducted using RStudio (version 4.3.1). Data from the NHANES 2011–2018 survey cycles were merged for analysis. Participants with missing data on variables required for each respective analysis were excluded using complete‐case analysis Descriptive statistics were presented as means with standard deviations for continuous variables. Latent class analysis was performed using the poLCA package, multinomial logistic regression using the nnet package, and Oaxaca‐Blinder decomposition using the Oaxaca package. Statistical significance was assessed at *p* value < 0.05.

## 3 Results

**FIGURE 1:**
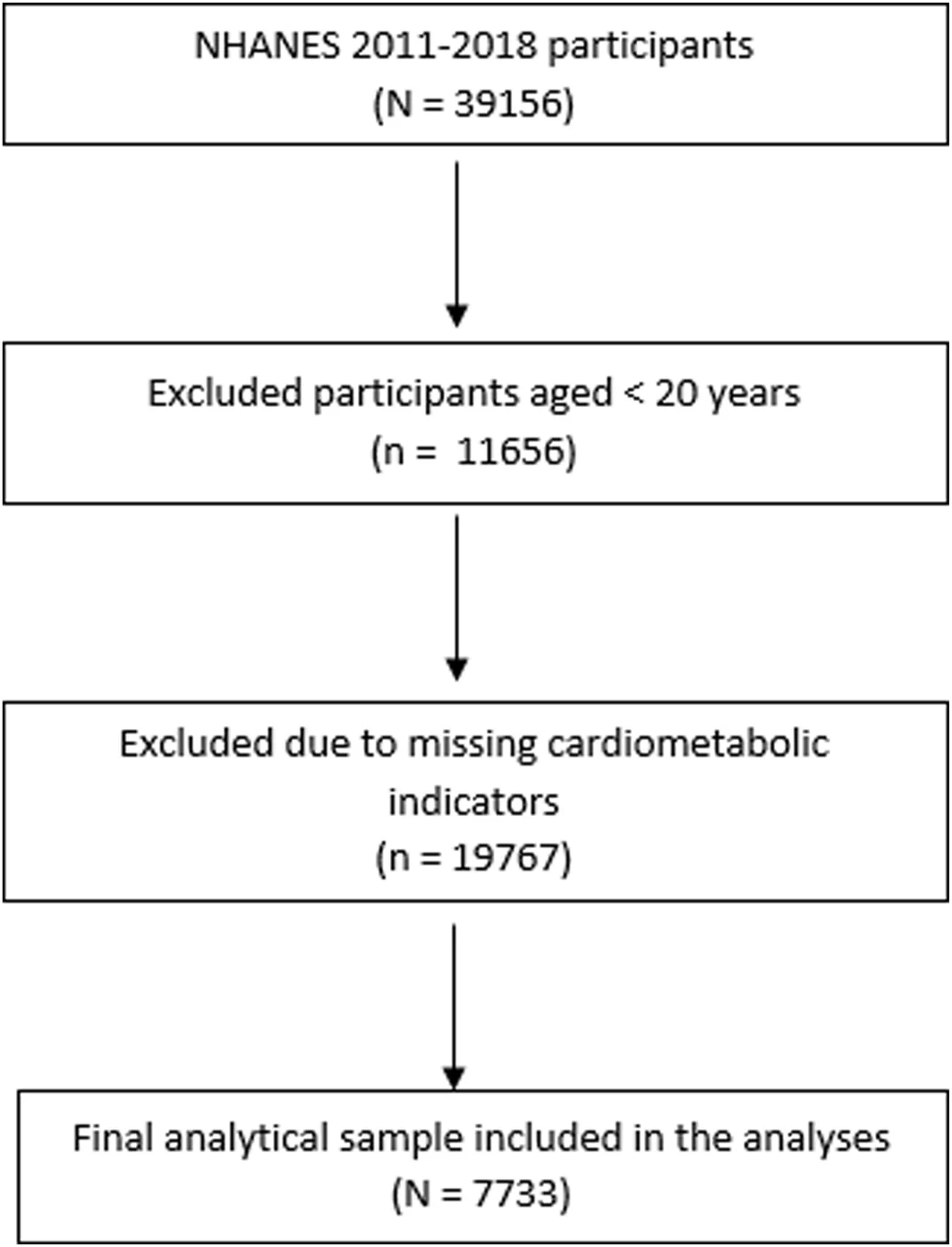
Flow diagram of the selection of participants.

A total of 39,156 participants from NHANES 2011–2018 were initially assessed for eligibility. Participants aged below 20 years were excluded (*n* = 11,656), leaving 27,500 adults eligible for analysis. Further exclusion of participants with missing cardiometabolic markers (*n* = 19,767) resulted in a final analytic sample of 7733 participants (Figure 1, Table 1). Six biomarkers reflecting major physiological components of cardiometabolic health‐waist circumference, systolic blood pressure, fasting plasma glucose, glycated haemoglobin (HbA1c), triglycerides, and high‐density lipoprotein (HDL) were examined to assess the underlying cardiometabolic structure prior to phenotype identification.

**TABLE 1:** Sociodemographic and behavioural characteristics of the study population.

Variable	Overall (*n* = 7733)
Age, mean (SD)	49.34 (17.4)
Sex, *n* (%)	
Female	3913 (50.6)
Male	3820 (49.4)
Race/Ethnicity, *n* (%)	
Mexican American	1023 (13.2)
Other Hispanic	810 (10.5)
Non‐Hispanic White	3061 (39.6)
Non‐Hispanic Black	1598 (20.7)
Other race	1241 (16.0)
Education, *n* (%)	
< 9th grade	643 (8.3)
9–11th grade	979 (12.7)
High school	1692 (21.9)
Some college	2407 (31.1)
College graduate	2009 (26.0)
Income‐to‐poverty ratio, mean (SD)	2.50 (1.63)
Low income, *n* (%)	
No	5263 (68.1)
Yes	2470 (31.9)
Current smoker, *n* (%)	
No	4327 (56.0)
Yes	3406 (44.0)
Physically inactive, *n* (%)	
No	1788 (23.1)
Yes	5945 (76.9)

Latent class analysis was consequently conducted using six binary cardiometabolic risk indicators derived from established clinical thresholds. Model fit was evaluated for latent class solutions ranging from two to four classes using Akaike Information Criteria (AIC), Bayesian Information Criteria (BIC), entropy, and class proportions. Both AIC and BIC decreased as the number of classes increased, indicating improved statistical fit with additional classes. The four‐class model produced the lowest AIC and BIC, however the three‐class model demonstrated the highest entropy (0.75), indicating better classification accuracy. Based on these considerations, the three class‐model was selected. The three‐class model produced a maximum log‐likelihood of −33,567.41, with Akaike Information Criterion (AIC) = 67,174.81 and Bayesian Information Criterion (BIC) = 67,319.71. The model estimated 20 parameters with 43 residual degrees of freedom. The likelihood ratio statistic was *G* ^2^ = 295.36, and the Pearson chi‐square goodness‐of‐fit statistic was 304.93, indicating an adequate representation of the observed data structure (Table 2). The three‐class solution demonstrated good classification quality, with average posterior probabilities of 0.829, 0.807, and 0.911 for the metabolically healthy, hypertension–hyperglycaemia, and high cardiometabolic risk phenotypes, respectively, indicating satisfactory certainty of class assignment (Supporting Information S1: Table S1).

**TABLE 2:** Fit statistics for latent class models with varying numbers of cardiometabolic phenotypes.

Number of classes	AIC^a^	BIC^b^	Entropy	Smallest class (%)
2	55,923.95	56,014.34	0.59	49.2
3	55,267.69	55,406.76	0.75	20.4
4	55,080.84	55,268.57	0.66	14.2

^a^ Akaike information criterion.

^b^ Bayesian information criterion.

The estimated class distribution indicated that 54.9% of participants belonged to a metabolically healthy phenotype, 33.1% belonged to an intermediate hypertension‐hyperglycemia dominant phenotype and 11.9% belonged to a high cardiometabolic risk phenotype (Supporting Information S1: Figure S1). Examination of conditional probabilities demonstrated clear distinctions across three latent cardiometabolic phenotypes. The metabolically healthy phenotype exhibited low probabilities of all metabolic abnormalities, including waist circumference (0.21), elevated glucose (0.25), elevated HbA1c (0.10), hypertriglyceridemia (0.10), low HDL cholesterol (0.06), and high blood pressure (0.17). The hypertension‐hyperglycemia phenotype was characterised predominantly by glycaemic and blood pressure abnormalities, with high probabilities of elevated glucose (0.86), elevated HbA1c (0.75), and high blood pressure (0.52), while lipid abnormalities remained relatively low. In contrast, the high cardiometabolic risk phenotype demonstrated the most adverse metabolic profile (0.81), hypertriglyceridemia (0.70) and low HDL cholesterol (0.83) (Supporting Information S1: Table S2).

Multinomial logistic regression analysis showed that age, sex, income, and education were significantly associated with cardiometabolic phenotype membership. Increasing age was associated with higher odds of both hyperglycemia‐hypertension phenotype (AOR: 1.07; 95% CI: 1.06–1.07) and the high cardiometabolic risk phenotype (AOR: 1.04; 95% CI: 1.03–1.04). Male population showed significantly greater odds of belonging to the high cardiometabolic risk phenotype (AOR: 4.00; 95% CI: 3.40–4.70). Significant racial and ethnic differences were also observed, with non‐Hispanic black and Mexican American participants demonstrating higher odds of membership in the hyperglycemia‐hypertension phenotype compared with non‐Hispanic White participants. Lower income and educational attainment were also significantly associated with a high cardiometabolic risk phenotype (Table 3).

**TABLE 3:** Multinomial logistic regression analysis of factors associated with cardiometabolic phenotype membership.

Variable	Hypertension‐hyperglycemia versus metabolically healthy OR	95% CI	High cardiometabolic risk versus metabolically healthy OR	95% CI
Age	1.07^a^	1.06–1.07	1.04^a^	1.03–1.04
Male sex	1.38^a^	1.23–1.54	4.00^a^	3.40–4.70
Mexican American	1.80^a^	1.50–2.16	1.23	0.97–1.56
Non‐Hispanic Black	1.93^a^	1.67–2.24	0.65^a^	0.51–0.82
Other Hispanic	1.36^a^	1.12–1.64	1.23	0.96–1.58
Other race	1.16	0.98–1.37	0.87	0.69–1.09
< 9th grade	1.53^a^	1.19–1.97	1.60^a^	1.14–2.24
9–11th grade	1.42^a^	1.15–1.74	1.55^a^	1.17–2.05
High school	1.47^a^	1.24–1.74	1.35^a^	1.06–1.72
Some college	1.37^a^	1.18–1.60	1.54^a^	1.24–1.91
Income to poverty ratio	0.96	0.93–1.00	0.88^a^	0.83–0.93

Abbreviations: CI, confidence interval; OR, odds ratio.

^a^ Statistically significant.

**FIGURE 2:**
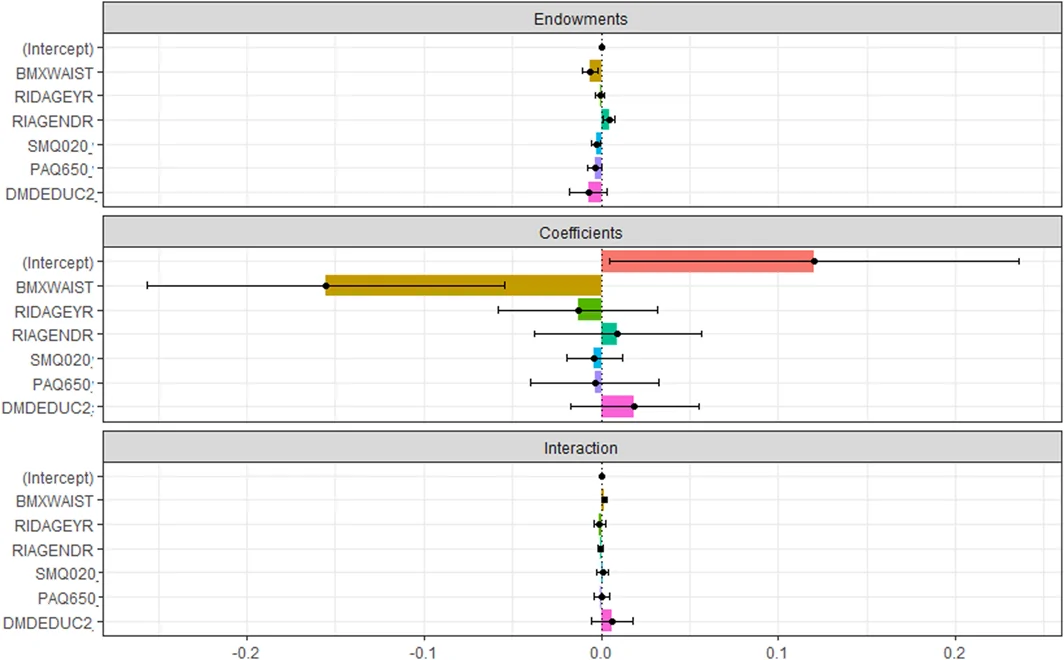
Oaxaca–Blinder decomposition of income‐related differences in the high cardiometabolic risk phenotype among US adults.

To further explore socioeconomic disparities in cardiometabolic health, participants were categorised into low income and high income group. Oaxaca‐Blinder decomposition analysis demonstrated a modest difference in the prevalence of the high cardiometabolic risk phenotype between the income groups, with slightly higher prevalence among higher‐income participants (15%) compared with lower‐income individuals (11.4%). Only a small proportion of the observed difference (1.2%) was explained by the measured characteristics (endowment component), whereas the majority of the disparity remained unexplained. A larger proportion of disparity was related to differences in the effect of these characteristics across groups (coefficient component), suggesting that socioeconomic inequalities in cardiometabolic risk may be influenced by broader structural or contextual factors beyond the measured variables alone. While waist circumference showed the largest negative contribution, variables such as education and sex demonstrated mall‐positive contributions, indicating a limited role in increasing the disparity. However, most confidence intervals crossed zero, suggesting that these contributions were not statistically robust. Overall, the income‐related differences in the high cardiometabolic‐risk phenotype were modest and only weakly explained by the measured sociodemographic and behavioural variables included in the model (Figure 2).

Age‐adjusted temporal analysis revealed notable shifts in the distribution of cardiometabolic phenotypes across NHANES cycles from 2011–2012 to 2017–2018. The prevalence of the metabolically healthy phenotype showed a declining trend over time, decreasing from approximately 52% in 2011‐12 to around 45% in 2017–2018. In contrast, the hyperglycemia‐hypertensive dominant phenotype demonstrated an overall increasing trend, rising from 36% to 43% across the study period. The high cardiometabolic risk phenotype remained relatively stable, with a slight increase from 11% to 13% observed between 2011–2012 and 2015–2016 followed by a marginal decline in 2017–2018. Despite this stability, the persistent presence of this high risk group indicates a sustained burden of cardiometabolic disease within the population (Supporting Information S1: Figure S2).

## 4 Discussion

In this nationally representative study of U.S. adults, distinct cardiometabolic phenotypes were identified using a person‐centred analytical approach. Three clinically meaningful groups emerged, including metabolically healthy phenotype, a hypertension‐hyperglycaemic phenotype, and a high cardiometabolic risk phenotype. A substantial proportion of individuals were classified within the hypertension‐hyperglycaemic group, highlighting the presence of an intermediate cardiometabolic phenotype characterised predominantly by abnormalities in glycaemic regulation and systolic blood pressure. Sociodemographic factors, including age, sex and socioeconomic indicators, were associated with phenotype membership although the magnitude of these associations varied. Decomposition indicated that only a modest proportion of income‐related differences in the high cardiometabolic risk phenotype was explained by the measured demographic, socioeconomic, anthropometric and behavioural characteristics, suggesting that additional unmeasured factors may contribute to the observed disparities.

The identification of distinct cardiometabolic phenotypes underscores the heterogenous nature of metabolic risk within the population. Rather than a simple binary distinction between healthy and unhealthy individuals, the findings of the study emphasises on the existence of an intermediate risk profile characterised by partial metabolic dysregulation. Beyond traditional metabolic syndrome definitions, the present findings demonstrate the added value of latent class analysis in identifying naturally occurring cardiometabolic phenotypes based on patterns of coexisting metabolic abnormalities rather than predefined diagnostic criteria. While the elected indicators overlap with conventional metabolic syndrome components, the person‐centred approach identified an intermediate hypertension‐hyperglycaemia phenotype characterised primarily by abnormalities in systolic blood pressure and glycaemic regulation without uniformly elevated lipid abnormalities. Such phenotypic distinctions may provide additional insight into population heterogeneity and support more targeted prevention strategies than conventional syndrome‐based classifications. These findings are also consistent with the recently proposed American Heart Association Cardiovascular‐Kidney‐Metabolic (CKM) health framework, which conceptualises cardiometabolic health as a continuum of interconnected metabolic abnormalities rather than a single syndrome. Although kidney function markers were not included in the present analysis, the identification of distinct cardiometabolic phenotypes supports the broader CKM concept that cardiometabolic exists across heterogenous metabolic profiles. The intermediate phenotype observed in this study is particularly important from a public health lens as it may represent a critical window for early intervention before progression to more severe metabolic abnormalities.

These findings are consistent with previous studies using latent class approaches, which have similarly identified heterogenous cardiometabolic profiles ranging from low risk to high risk clusters. Prior research has demonstrated that such phenotypic classifications may provide greater insight into disease risk rather than traditional definitions which rely on predefined thresholds. However, the distribution and characteristics of identified classes vary across populations, reflecting differences in demographic composition, lifestyle factors, and environmental exposures. The present study contributes to the large body of literature by examining these patterns in a large number of U.S. adults participating in NHANES 2011–2018 and by integrating socioeconomic analysis in the phenotyping framework.

The observed associations between phenotype membership and measured sociodemographic characteristics highlight the multifactorial nature of cardiometabolic health. Increasing age and male sex were consistently associated with higher odds of adverse cardiometabolic phenotypes, reflecting well‐established epidemiological patterns. Significant differences were observed across racial and ethnic groups. These findings should not be interpreted as evidence of inherent biological differences between populations. Rather, they likely reflect the influence of demographic and socioeconomic circumstances and may also be shaped by broader contextual, environmental and healthcare‐related factors that were not directly measured in the present study. Similarly, income and educational attainment represent important socioeconomic indicators but do not fully capture the broader structural determinants of health that contribute to cardiometabolic disparities.

The observed racial/ethnic and socioeconomic differences are consistent with the broader cardiovascular health disparities. Disparities in cardiovascular health arise through complex interactions among socioeconomic position, healthcare access, neighbourhood environment, and other social determinants rather than inherent biological differences [6]. Similarly, structural and environmental factors substantially influence the cardiovascular risk and contribute to persistent health problems [5]. Accordingly, the associations observed in the present study should be interpreted within this broader context and not as evidence of intrinsic biological susceptibility among different population groups.

The Oaxaca–Blinder decomposition analysis was performed to complement the latent class analysis by quantifying the extent to which measured characteristics explained income‐related differences in the high cardiometabolic risk phenotype. Only a modest proportion of the observed disparity was explained by the variables included in the model, while the majority remained unexplained. These findings suggest that additional contextual, environmental, healthcare‐related, or structural factors not captured in the present analysis may contribute to the observed socioeconomic differences The family income‐to‐poverty ratio (INDFMPIR) provides a standardised measure of socioeconomic position within NHANES and was therefore considered an appropriate indicator for examining socioeconomic differences in cardiometabolic phenotype distribution. Central obesity emerged as the largest negative contributor to inequality, indicating that the relationship between adiposity and cardiometabolic risk differed across socioeconomic groups in a manner that reduced the observed disparity. This finding may reflect the complex and non‐linear association between obesity and socioeconomic status within the US population, where elevated adiposity is prevalent across both lower and higher income groups. In contrast, educational attainment and sex demonstrated small positive contributions suggesting limited role in widening socioeconomic differences in high cardiometabolic risk phenotype. Smoking and physical inactivity demonstrated minimal contributions, suggesting that behavioural differences alone does not fully explain the observed socioeconomic inequalities in cardiometabolic phenotypes. Consequently, these findings should be interpreted cautiously rather than as definitive evidence regarding the mechanisms underlying socioeconomic differences in cardiometabolic phenotypes.

The observed temporal trends indicate a declining proportion of metabolically healthy individuals accompanied by an increasing prevalence of the intermediate hypertension‐hyperglycemia phenotype. This pattern may reflect broader epidemiological changes, including increasing obesity prevalence, sedentary lifestyles, and dietary transitions over time. This pattern suggests that an increasing proportion of U.S. adults exhibit concurrent abnormalities in blood pressure and glycaemic regulation, underscoring the importance of early preventive strategies.

The study has several strengths. The use of a large nationally representative dataset (NHANES) enhances the generalisability of findings. The application of LCA allowed for the identification of clinically meaningful cardiometabolic phenotypes based on real‐world clustering patterns. Furthermore, the integration of multiple analytical approaches, including regression, decomposition methods and trend analysis provides a comprehensive assessment of both determinants and socioeconomic inequalities in cardiometabolic risk.

The limitations of the study must be acknowledged. The cross‐sectional design of NHANES precludes causal interference and does not permit assessment of temporal relationships between the identified cardiometabolic phenotypes, which should therefore be interpreted as cross‐sectional groupings rather than stages of disease progression. Second, a complete‐case analysis was performed because latent class analysis requires complete observations for all indicator variables, and the exclusion of participants with missing data may have introduced selection bias. Third, the analyses did not incorporate the survey weights, strata and primary sampling units as software packages used for the analyses do not readily accommodate the complex NHANES survey design potentially limiting the national representativeness of the findings. Furthermore, important aspects of cardiometabolic health, including medication use, kidney function, and other cardiovascular risk factors, were not included in the latent class model. Finally, continuous biomarkers were dichotomised using established clinical thresholds to facilitate latent class analysis, which may have reduced information on obscured variability within risk categories. These limitations should be considered while interpreting the study findings. The limitations of the study must also be acknowledged.

The findings of the study have important implications for public health. The identification of an intermediate hypertension‐hyperglycaemic phenotype highlights the potential value of early detection and targeted preventive interventions among individuals exhibiting early metabolic dysregulation prior to progression to overt cardiometabolic disease. Furthermore, the observed contributions of obesity, education, and socioeconomic strategies should extend beyond income‐based approaches and incorporate broader structural and metabolic determinants of health.

## 5 Conclusion

Cardiometabolic risk in the U.S. population clusters into distinct phenotypes, including an intermediate hypertension‐hyperglycemia phenotype associated with substantial metabolic burden. Several sociodemographic and metabolic factors were associated with phenotype membership, highlighting the heterogeneity of cardiometabolic risk. These findings demonstrate the utility of a patient‐centred approach for characterising cardiometabolic heterogeneity and may help inform future risk stratification and targeted preventive strategies. Further longitudinal studies are warranted to validate these phenotypes and explore their clinical implications.

## Author Contributions

Greeshma Unnikrishnan conceptualized the study, performed data analysis, interpreted the findings, and drafted the manuscript. Abhinav Singh contributed to study supervision, methodological guidance, interpretation of results, and critical review of the manuscript. K. R. Harijith contributed to data interpretation, and manuscript review. All authors have read and approved the final version of the manuscript.

## Funding

The authors have nothing to report.

## Conflicts of Interest

The authors declare no conflicts of interest.

## Supporting information


Supporting Information S1

## Data Availability

The data that support the findings of this study are openly available in National Health and Nutrition Examination Survey (NHANES) at https://www.cdc.gov/nchs/nhanes‐participants/index.html.
