# Supplementary material for: Latent Cardiometabolic Phenotypes and Their Sociodemographic Correlates Among US Adults: A Cross‐Sectional Latent Class Analysis Using NHANES (2011–2018) Data
Source: Diabetes Metab Res Rev. 2026 Aug 31;42(6):e70225. doi: 10.1002/dmrr.70225 (PMC13528256; doi:10.1002/dmrr.70225)
Supplement: Supplementary file 1 [file DMRR-42-e70225-s001.docx]

**SUPPLEMENTARY APPENDIX**

**Figure 1: Radar plot of latent cardiometabolic phenotypes (NHANES 201-18)**

**Figure 2: Age-adjusted prevalence of latent cardiometabolic phenotypes across NHANES cycles- A trend analysis**

**Table 1 : Classification quality of the selected three-class latent class model**

| **Latent Class** | **Phenotype** | **Average Posterior Probability** |
| --- | --- | --- |
| Class 1 | Metabolically Healthy | 0.829 |
| Class 2 | Hypertension–Hyperglycaemia | 0.807 |
| Class 3 | High Cardiometabolic Risk | 0.911 |

**Table 2. Conditional probabilities of cardiometabolic abnormalities across latent classes**

| **Cardiometabolic Indicator** | **Metabolically healthy phenotype** | **Hypertension-hyperglycaemia phenotype** | **High cardiometabolic risk phenotype** |
| --- | --- | --- | --- |
| High waist circumference | 0.21 | 0.56 | 0.69 |
| High glucose | 0.25 | 0.86 | 0.81 |
| High HbA1c | 0.10 | 0.75 | 0.58 |
| High triglycerides | 0.10 | 0.22 | 0.70 |
| Low HDL cholesterol | 0.06 | 0.05 | 0.83 |
| High blood pressure | 0.17 | 0.52 | 0.37 |
